# A Poly-D-lysine-Coated Coralline Matrix Promotes Hippocampal Neural Precursor Cells’ Differentiation into GFAP-Positive Astrocytes

**DOI:** 10.3390/polym15204054

**Published:** 2023-10-11

**Authors:** Roni Mina Hendler, Orly Eva Weiss, Tzachy Morad, Guy Sion, Michael Kirby, Zvy Dubinsky, Ayan Barbora, Refael Minnes, Danny Baranes

**Affiliations:** 1 Department of Molecular Biology, Ariel University, Ariel 4070000, Israel; 2 Department of Science, The David Yellin Academic College of Education, Jerusalem 9103501, Israel; 3 Adelson School of Medicine, Ariel University, Ariel 4070000, Israel; 4 The Mina & Everard Goodman Faculty of Life Sciences, Bar-Ilan University, Ramat-Gan 5290002, Israel; 5 Department of Physics, Ariel University, Ariel 4070000, Israel

**Keywords:** polylysine, calcium carbonate, hippocampal cells, neural progenitor cells, astrocytes, nestin, glial fibrillary acidic protein

## Abstract

A major goal of regenerative medicine of the central nervous system is to accelerate the regeneration of nerve tissue, where astrocytes, despite their positive and negative roles, play a critical role. Thus, scaffolds capable of producing astrocytes from neural precursor cells (NPCs) are most desirable. Our study shows that NPCs are converted into reactive astrocytes upon cultivation on coralline-derived calcium carbonate coated with poly-D-lysine (PDL-CS). As shown via nuclei staining, the adhesion of neurospheres containing hundreds of hippocampal neural cells to PDL-CS resulted in disaggregation of the cell cluster as well as the radial migration of dozens of cells away from the neurosphere core. Migrating cells per neurosphere averaged 100 on PDL-CS, significantly higher than on uncoated CS (28), PDL-coated glass (65), or uncoated glass (20). After 3 days of culture on PDL-CS, cell migration plateaued and remained stable for four more days. In addition, NPCs expressing nestin underwent continuous morphological changes from round to spiky, extending and elongating their processes, resembling activated astrocytes. The extension of the process increased continuously during the maturation of the culture and doubled after 7 days compared to day 1, whereas bifurcation increased by twofold during the first 3 days before plateauing. In addition, nestin positive cells’ shape, measured through the opposite circularity level correlation, decreased approximately twofold after three days, indicating spiky transformation. Moreover, nestin-positive cells co-expressing GFAP increased by 2.2 from day 1 to 7, reaching 40% of the NPC population on day 7. In this way, PDL-CS promotes NPC differentiation into reactive astrocytes, which could accelerate the repair of neural tissue.

## 1. Introduction

The complexity of neuronal networks and the weakness of the regenerative capacity of nervous tissue make regenerative medicine of the damaged central nervous system especially challenging. Consequently, tissue engineering approaches for wound healing and tissue restoration are needed for curing neurological tissue injuries. Biomaterial scaffolds made from biocompatible and non-toxic substances are the most popular among these. Biocompatible polymers have attracted significant attention in the realm of regenerative medicine, because of their unique properties and chemical versatility. Cationic polymers have often been used in biomedical applications because of their interaction with negatively charged biomolecules, peptides, proteins, and nucleic acids. The use of polycationic polymers in cell membranes is widespread for transporting cargoes such as drugs and genes and for improving cell–substrate interactions and adhesion [1,2]. Poly(amino acid)s are cationic polymers that have several advantages in biomedicine, including biocompatibility, bioactivity, and nontoxicity [3,4]. Consequently, poly(amino acid) has been used as a substrate for the growth and differentiation of various cell and tissue types [5,6,7,8].

Polylysine is a cationic poly(amino acid)s which has attracted significant attention in tissue engineering because of its biodegradability and biocompatibility. Due to its positively charged nature, polylysine easily interacts with negatively charged surfaces on many types of cells [9,10]. Poly-D-lysine (PDL), a synthetic homopolyamide of the amino acid lysine has been found to be highly supportive of tissue regeneration and has long been used for the culture of different cells types [11], including neural cells, promoting their adhesion, survival, growth, and guidance of hippocampal, cortical, and cerebral neurons and glial cells [12,13,14,15,16].

PDL has also been applied as a coating substance for increasing the bioregenerative efficiency of nanoparticles and biomaterials [11,17,18,19], resulting in stable and prolonged cell adhesion because of its positively charged hydrophilic properties [20]. For instance, PDL has been shown to bind directly to skeletons of coral [20]. PDL-coated coral skeletons (PDL-CS) enhance the CS effect on the survival, growth, connectivity, and activity of hippocampal neurons in dissociated cultures compared to PDL-coated glass [20,21,22,23,24,25,26,27].

In addition to its positive role in neuronal growth and survival, PDL-CS increases the activity of hippocampal astrocytic cells as indicated by the increased expression of their cytoskeletal protein Glial Fibrillary Acidic Protein (GFAP) as well as a shift in morphology from flat and smeared nascent cells to spiky [20,22,23,26]. The positive effect of PDL-CS on astrocytic shape and activity suggests that it causes astrogliosis, in which nascent astrocytes become reactive astrocytes, an essential part of the regeneration process after brain injury [28,29]. The release of neurotrophic factors and cytokines by reactive astrocytes plays an important role in regulating neuronal function in the brain [30,31]. These secreted molecules can enhance cell survival, re-myelination, suppress the inflammatory response, and encourage the migration and differentiation of neural precursor cells (NPCs) at the site of injury, which differentiate into astrocytes and promote astrogliosis [32,33,34,35].

Due to astrocytes’ regenerative function, the accumulation of astrocytes near a wound is fundamental to its recovery, so any method that generates astrocytes via the differentiation of NPCs, attracts them to the injury site, and guides them to the wound would be extremely beneficial for the recovery of damaged nerve tissue and astrogliosis. But, neither PDL nor CS have been reported to affect the differentiation of NPCs to astrocytes. Therefore, the purpose of this study was to test if PDL-CS influences the parameters that can increase the number of reactive astrocytes at a wound, such as the migration of NPCs, their differentiation into GFAP-expressing astrocytes, and their activation.

We show in this study that PDL-CS promotes the migration of nestin-positive NPCs, elongates their processes, and induces them to become GFAP-expressing cells, suggesting that it stimulates their differentiation into reactive astrocytes. As a result, PDL-CS may be useful in promoting recovery from central nervous system injuries by enhancing astrogliosis.

## 2. Materials and Methods

### 2.1. Preparation of the Coral Skeleton

The exoskeletons of Trachyphyllia geoffroyi were sectioned into 0.2–1.0 mm sections and treated with sodium hypochlorite solution (10%, RT, 10 min). Sigma-Aldrich 425044, Burlington, MA, USA) and NaOH solution (1 M, 5 min, RT, Sigma Aldrich S8045) were used to digest any adhering organic matter. The fragments were then transferred to a H_2_O_2_ solution (30% *v*:*v* aq, 10 min, RT, Romical, Be’er Sheva, Israel). Afterward, the fragments were rinsed with distilled water, air-dried, and ground using a Smart Dentin Grinder (KometaBio, Cresskill, NJ, USA). Following that, grains sized under 40 mm were sieved.

### 2.2. Coating Coverslips with CS and PDL

A suspension of sieved CS grains was prepared in double distilled water (DDW, 25 mg CS grains per mL). The solution was dispersed onto coverslips (12 mm, Menzel-Glaser, 100 mL/coverslip) and dried at 80 °C. After autoclaving, the coated coverslips were stored at RT until use. Prior to culture, coated coverslips were coated with 100 mL PDL (30–70 kDa, 20 g/mL; A-003-M, Sigma-Aldrich, USA) overnight at 4 °C. Before culturing, PDL-coated coverslips were washed with DDW and dried in a hood.

### 2.3. Preparation of Neurospheres

Preparation of neurospheres began with the extraction of neural cells from postnatal rats’ hippocampi. The cells were obtained from hippocampi of 1–2 day-old rat pups, according to Baranes et al. [36]. The hippocampi were dissected out of the brain and dissociated using trypsin (0.25%, 30 min, 37 °C; Sigma-Aldrich, USA). Using pasture pipets, the tissue was triturated in Minimal Essential Eagle’s Medium (87%, Sigma-Aldrich, USA), heat-inactivated fetal bovine serum (10%, Sigma-Aldrich, USA), D-glucose (2%, ThermoFisher Scientific, Waltham, MA, USA), and L-glutamine (1%, ThermoFisher Scientific, Waltham, MA, USA).

Cells were arranged into neurospheres in accordance with Morad et al. [37]. Briefly, the primary neural cells were resuspended in proliferation media containing Minimal Essential Eagle’s Medium (45%, Sigma-Aldrich, USA), Dulbecco’s Modified Eagle Medium (40%, Sigma-Aldrich, USA), Ham’s Nutrient Mixture F-12 (10% *w*:*v*, Sigma-Aldrich, USA), D-glucose (0.75%, ThermoFisher Scientific, USA), B-27 (0.5%, ThermoFisher Scientific, USA), bovine serum albumin (0.25%, Sigma-Aldrich, USA), L-glutamine (0.25%, ThermoFisher Scientific, USA), and epidermal growth factor (20 ng/mL; ThermoFisher Scientific, USA). After being plated in 24-well plates at 105/mL^−1^, the cells were incubated at 37 °C with 10% CO_2_ for one week.

The cells accumulated into neurospheres, each containing hundreds of cells. Each coverslip was loaded with 20–40 neurospheres in 100 µL of supplemented media consisting of Minimal Essential Eagle’s Medium (45%, Sigma-Aldrich, USA), Dulbecco’s Modified Eagle Medium (40%, Sigma-Aldrich, USA), Ham’s Nutrient Mixture F-12 (10%, Sigma-Aldrich, USA), D-glucose (0.75%, ThermoFisher Scientific, USA), B-27 (0.5%, ThermoFisher Scientific, USA), bovine serum albumin (0.25%, Sigma-Aldrich, USA), L-glutamine (0.25%, ThermoFisher Scientific, USA), kynurenic acid (0.01%, Sigma-Aldrich, USA), and 0.01% of an anti-mitotic composed of 70% uridine (Sigma-Aldrich, USA) and 30% fluoro-deoxy-uridine (Sigma-Aldrich, USA). Cultures were incubated overnight at 37 °C with 10% CO_2_. On the following day, 0.5 mL of supplemented culture medium was added to each well and the cells were incubated at 37 °C, 10% CO_2_, for 1, 3, and 7 days.

### 2.4. Immunofluorescence

The neurospheres were fixed with 4% paraformaldehyde (10 min, RT, Sigma-Aldrich, USA), permeabilized with 0.25% Triton X-100 (5 min, RT, TEDIA, Fairfield, OH, USA), and blocked for 1 h (RT, 3% inactivated normal goat serum plus 0.1% Triton X-100). The samples were then incubated (overnight, 4 °C) with rabbit IgG polyclonal antibodies raised against GFAP and an IgG mouse monoclonal antibody raised against nestin protein (Abcam, Cambridge, UK), followed by secondary goat anti-rabbit IgG (Millipore, Burlington, MA, USA) and goat anti-mouse IgG (Millipore, USA). Nuclei were stained with 4′,6-diamidino-2-phenylindole (DAPI, Sigma, USA). Fluoromount containing 2.5% 1,4-diazabicyclo[2.2.2]octane (DABCO, Sigma, USA) was used for mounting.

### 2.5. Image Analyses

The fluorescent images were acquired using an inverted Zeiss Axio-observer Z1 (Zeiss, Southern Germany) microscope equipped with an objective of X10/0.3 and fluorescent DAPI, FITC, and Rhodamine filter cubes. This image analysis was performed with ImageJ using the Fiji package [4], an open-source platform for biological-image analysis (https://imagej.net/software/fiji/ (accessed on 1 November 2022)).

After manually removing the neurosphere core from the images, we counted the cells using the “Find Maxima” function on the DAPI channel (stains nuclei). The migration distances were measured by manually marking the distance from the core using the segmented line tool. The branch length was measured by adjusting the threshold to show outlines of branches. The images were then subjected to Sholl analysis using the default parameters in the dialog box. With the “Wand” tool and “Shape Descriptors” measurement, cell branches were individually selected and measured for circularity. The program calculates circularity by dividing area by perimeter; 1.0 indicates a perfect circle and values approaching 0 indicate increasingly elongated polygons. Lastly, we measured colocalization after removing the neurosphere cores. Colocalization analysis was conducted using the “Colocalization Threshold” function, using the default parameters in the dialog box. To determine the level of colocalization, we used %Volume. The %Volume represents the percentage of pixels in the image with both red and green intensities above the threshold.

### 2.6. Statistics

The data are expressed as means (±SD), α = 0.05. The multiple comparison analyses were carried out either using one-way ANOVA followed by Fisher’s Least Significant Difference test for parametric analyses with normal distributions or using Kruskal–Wallis ANOVA followed by a Dunn’s test for nonparametric analyses. GraphPad Prism 9.0 was used for all the statistical analyses, and group differences were considered statistically significant when *p* ≤ 0.05 was achieved (* ≤0.05, ** ≤0.01, *** ≤0.001).

## 3. Results

**Figure 1:**
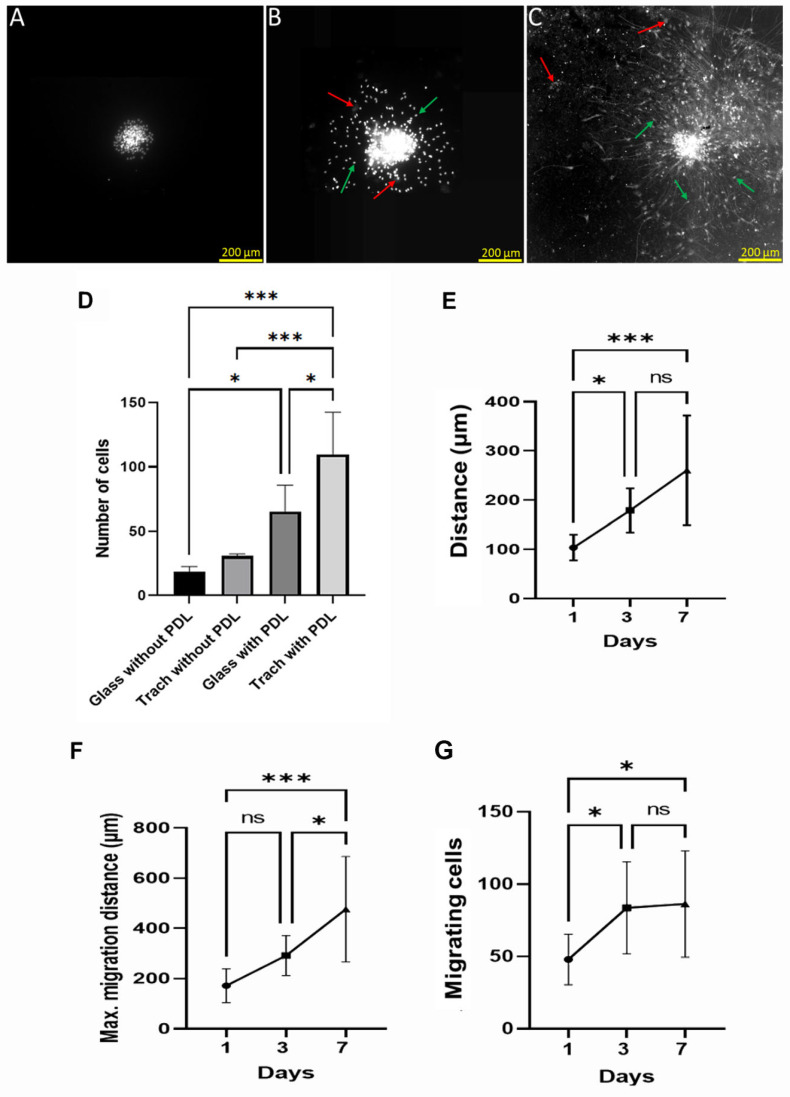
Activation of cell migration by attaching neurospheres to coral skeletons. These images show cell nuclei in (**A**) 1-, (**B**) 3-, and (**C**) 7-day-old cultures. Green arrows = DAPI; red arrows = coral skeleton grains. (**D**) Nuclei counting (4–7 days old) on glass and CS, coated or uncoated with poly-D-lysine. (**E**) cell migration distance. (**F**) Maximum distance for cell migration. (**D**–**F**) One-way ANOVA followed by Fisher’s Least Significant Difference (*, *p* ≤ 0.05; ***, *p* ≤ 0.001; ns, *p* > 0.05). (**G**) number of migrating cells. Kruskal–Wallis ANOVA followed by Dunn’s test (*p* < 0.05): *, *p* ≤ 0.05; ***, *p* ≤ 0.001; ns, *p* > 0.05.

Upon laying hippocampal neurospheres on PDL-CS substrate, radial cell outgrowth and spreading progressed from the first day of culture through to the seventh day (Figure 1A,B). In the days following the onset of culture, the neurosphere structure disaggregated (Figure 1B,C), with neurosphere cells dispersed far from the origin by day 7. As shown in Figure 1D, PDL on the CS promoted cell migration, as cell migration on PDL-CS was higher than that on PDL-depleted CS (*p* < 0.05). As migration progressed, the distances the migrating cells covered increased linearly (*p* ≤ 0.05 and *p* ≤ 0.001, respectively; Figure 1E,F). According to the cell-number-to-culture-age analysis, most cells migrated within 3 days with little change in migration thereafter (*p* ≤ 0.05; Figure 1F).

**Figure 2:**
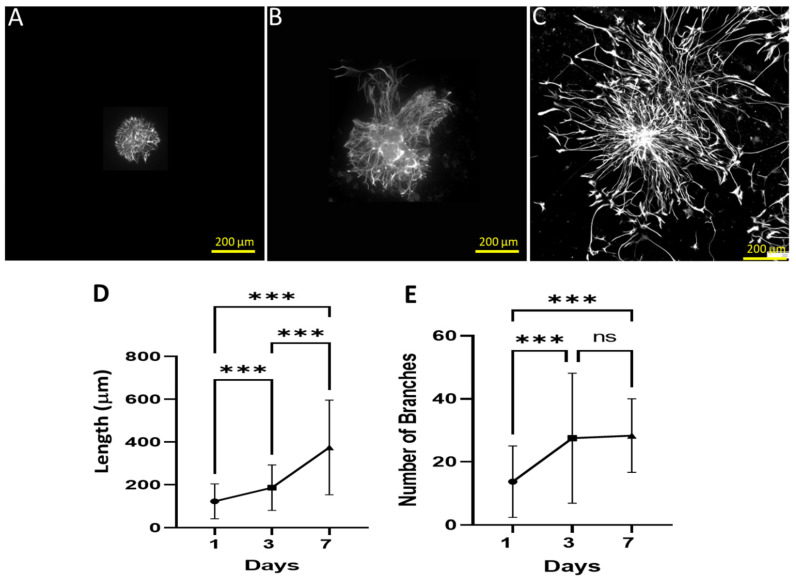
Nestin^+^ NPCs extend and ramify processes on PDL-CS. Anti-nestin antibody-stained cells at 1 (**A**), 3 (**B**), and 7 days of culture (**C**). The data are presented as the mean (±SD) of (**D**) process length and (**E**) number of branches. (**D**) One-way ANOVA followed by Fisher’s Least Significant Difference (*p* < 0.0001); (**E**) Kruskal–Wallis ANOVA followed by Dunn’s test (*p* ≤ 0.001): ***, *p* ≤ 0.001; ns, *p* > 0.05.

In the neurospheres, nestin-positive neural precursor cells (NPCs) made contact with PDL-CS by spreading their processes and migrating outward, resulting in markedly different patterns of cell dispersal and morphological differentiation (Figure 2A–C). Early morphological changes in neurosphere cultures were evident from their filamentous appearance on day 1 (Figure 2A). There was an increase in ramified nestin+ cell dispersion as migration and differentiation progressed (Figure 2B,C). Over time, the mean process length increased in a non-linear and progressive manner (*p* ≤ 0.001; Figure 2D). Similarly, nestin^+^ cell morphology with respect to branch processes appeared to progress from days 1 to 3, but to remain constant from days 3 to 7 (*p* ≤ 0.001; Figure 2E).

**Figure 3:**
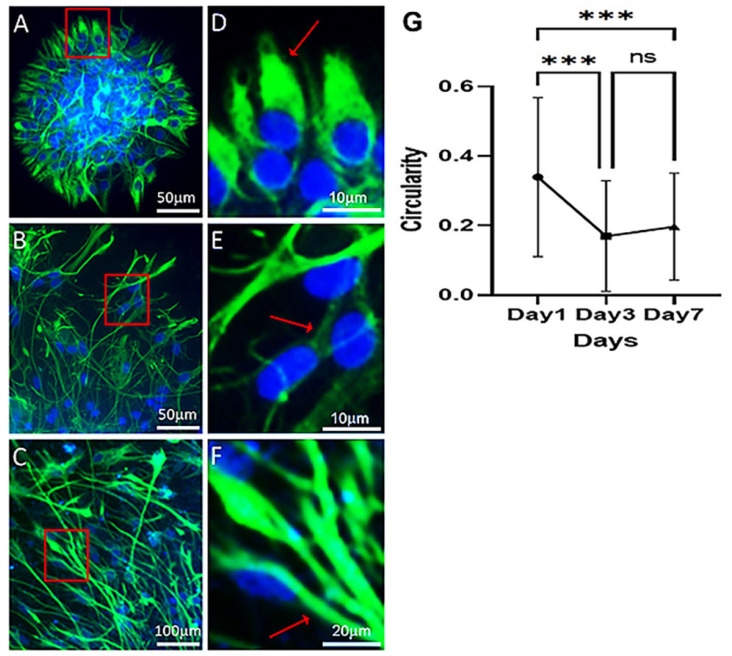
Nestin^+^ NPCs acquire an astrocytic shape on PDL-CS. DAPI (blue) and anti-nestin antibody (green) stained cells at (**A**,**D**) 1, (**B**,**E**) 3, and (**C**,**F**) 7 days. (**D**–**F**) Magnification of the red-framed area (**A**,**B**,**C**, respectively). A cell with a different circularity is indicated by an arrow. (**G**) Circularity of cells (mean (±SD)). One-way ANOVA followed by Fisher’s Least Significant Difference test (*p* ≤ 0.001). ***, *p* ≤ 0.001; ns, *p* > 0.05.

The nestin^+^ NPCs on PDL-CS gradually became spiky and filamentous, similar to activated astrocytes (Figure 3B,C). As shown in Figure 3D–F, nestin was clearly expressed in the cytoplasm and processes of the cells. A spiky appearance developed during culture maturation and plateaued at 3 days (*p ≤* 0.001; Figure 3G).

**Figure 4:**
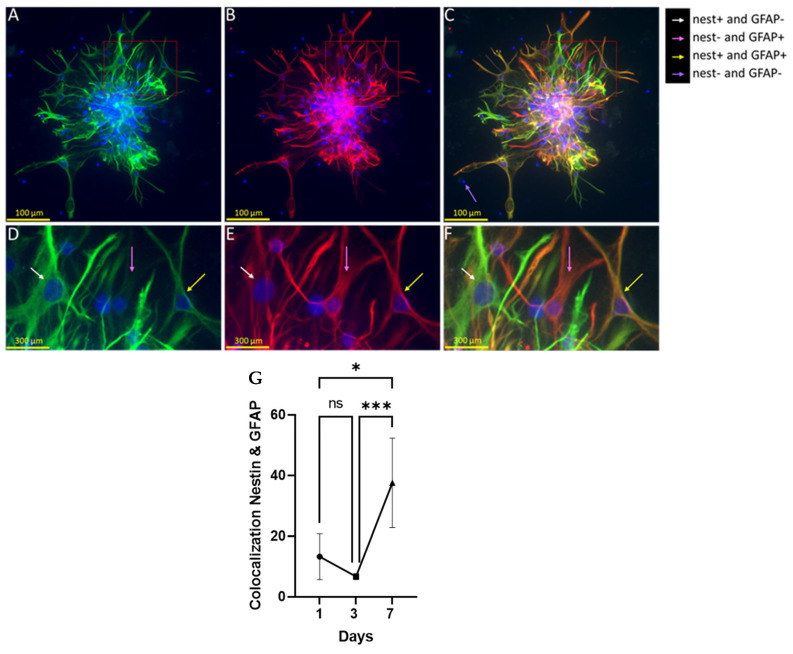
GFAP and nestin colocalization in NPCs grown on PDL-CS. The following are images of cells stained with (**A**) anti-GFAP antibody (green), (**B**) anti-nestin antibody (red), and (**A**,**B**) DAPI (blue) at day 7 of culture. (**C**) Merged image of (**A**,**B**) (yellow indicates nestin/GFAP colocalization). (**D**–**F**) Magnification of the red-framed area (of **A**,**B**,**C**, respectively). (**G**) A plot of the correlation between GFAP/nestin expression (% yellow/total (red + green) pixels). Kruskal–Wallis ANOVA followed by Dunn’s test (*p* ≤ 0.001). *, *p* ≤ 0.05; ***, *p* ≤ 0.001; ns, *p* > 0.05.

The colocalization analysis of cultures stained with anti-nestin and anti-GFAP antibodies was conducted to determine whether nestin^+^ NPC differentiation on PDL-CS was indeed directed towards glial lineage. In day 7 cultures, both cell subtypes were present and outgrew their processes (Figure 4A–C). Both proteins were highly expressed in the cell processes (Figure 4D,E). A portion of the processes expressed either one of the proteins, but there were also processes expressing both (Figure 4F). During culture aging, nestin/GFAP colocalization increased by more than twofold, particularly between days 3 and 7.

## 4. Discussion

Our study demonstrates that PDL-CS promotes hippocampal NPC differentiation towards glial lineage by enhancing their motility, ramifications, and GFAP expression within their processes. To perform this differentiative function effectively, PDL-CS must combine calcium carbonate together with a polymer coating. Based on the findings of this study, PDL-CS may serve as a scaffold for the regeneration of nervous tissue by stimulating the generation of reactive astrocytes from NPCs.

PDL-CS’s strength as a tissue regenerator can be assessed by measuring the extent to which cells are dissociated from the neurospheres. The cell clusters mainly dissociated during the first 3 days, and the size of the clusters did not decrease significantly thereafter (Figure 1C vs. Figure 1A), suggesting that only part of the cells detached. Based on a calculated average of 200 cells/neurosphere [24], we estimate this portion to be 5–10%. The partial cell detachment could be due to tight cell–cell interactions in neurospheres [26,27].

As a cell motility enhancer, PDL-CS is most effective because migrating cells on PDL-CS have longer migration distances than cells on uncoated CS or on PDL-coated or -uncoated glass (Figure 1C). As motility was measured using nuclei staining of the entire population, the enhanced migration on PDL-CS might have been due to an increase in proliferation. However, this possibility is unlikely, as migrating distances and numbers plateaued after 3 days on PDL-CS (Figure 1G). This idea is contradictory to the fact that PDL-CS has been found to stimulate cell proliferation in dissociated hippocampal cultures [19]. Since migration and proliferation are reciprocally regulated [28], PDL-CS might induce the migration of NPCs while reducing their proliferation in hippocampal neurosphere cultures.

The strong and continuous promotion of process growth is another regenerative strength of PDL-CS. In the 7 days of measurement, the NPCs’ morphological reaction to this matrix was linear, manifested by a continuous elongation of their processes. It is possible, however, that the elongation that was observed on the first day was overestimated due to cell migration, as similar migration and process length values were observed (approximately 100 μm). Additionally, the process-branching and overall spiking of NPCs were maximal at day 3, suggesting that they were related to cell migration. Several studies have confirmed this relationship between cell shape and migration [29,30,31]. From day 3 to 7, however, elongation continued to increase but migration did not, suggesting that NPCs’ contact with PDL-CS is the cause of process elongation rather than migration. Accordingly, PDL-CS promotes migration, promotes neural cell growth and ramification, and transforms NPCs to a spiky configuration similar to reactive astrocytes. In addition, growing NPCs on PDL-CS differentiated them to express GFAP, as revealed by nestin-GFAP colocalization measurements (Figure 4). Interestingly, colocalization increased between days 3 and 7, but not before, indicating that PDL-CS triggers process growth and branching, as well as cell migration, before elevating GFAP. GFAP expression can also indicate PDL-CS’ ability to increase astrocytic reactivity as discussed here and in other studies when primary hippocampal astrocytes were activated on PDL-CS [25,32]. A similar response of astrocytic cells is seen following TBI [33,34] as well as ischemia [35].

Based on the findings of this study, PDL-CS appears to increase differentiation through additive action with CS. PDL has demonstrated an inductive capacity by promoting the differentiation of embryonic stem cells into neurons [36]. Moreover, PDL has a similar structure to poly-L-lysine, so it may have a similar effect—increasing differentiation-related genes [37] and promoting the differentiation of stem cells [38,39,40] as well as NPCs [36]. As for coralline aragonite, it has been found to promote the differentiation of mesenchymal stem cells [41,42]. The effect of PDL and CS on differentiation may, therefore, be synergized when they are linked.

Thus, PDL-CS promotes the migration and differentiation of NPCs, which is crucial for wound healing in the central nervous system. By implanting PDL-CS grafts into brain wounds, NPCs can migrate, differentiate into astrocytes, and increase the astrogliosis process, leading to tissue regeneration, though scarring is also possible. 

## Data Availability

The data presented in this study are available upon request from the corresponding author.

## Abbreviations

NPCs	Neural Precursor Cells
PDL	Poly-D-lysine
CS	Coral Skeleton
PDL-CS	PDL-coated Coral Skeleton
GFAP	Glial Fibrillary Acidic Protein
